# The Investigation of Graphene Oxide-Enhanced Hybrid Slurry Preparation and Its Polishing Characteristic on CVD Single Crystal Diamond

**DOI:** 10.3390/ma17246053

**Published:** 2024-12-11

**Authors:** Zixuan Wang, Yang Zhao, Jie Yao, Tianbiao Yu, Sheng Qu, Jun Zhao

**Affiliations:** 1 School of Mechanical Engineering and Automation, Northeastern University, Shenyang 110819, China; wangzx@mail.neu.edu.cn (Z.W.);; 2 Liaoning Provincial Key Laboratory of High-End Equipment Intelligent Design and Manufacturing Technology, Northeastern University, Shenyang 110819, China; 3 School of Mechanical Engineering, Shenyang University of Technology, Shenyang 110870, China; 4 College of Mechanical Engineering, Zhejiang University of Technology, Hangzhou 310023, China

**Keywords:** single crystal diamond, polishing surface quality, material removal rate, graphene oxide

## Abstract

As an environment-friendly material, graphene oxide nanosheet can effectively improve the polishing surface quality of single crystal diamond workpieces. However, the lubricating and chemical effects of graphene oxide nanosheets have an uncertain impact on the polishing material removal rate. In this paper, the graphene oxide-enhanced hybrid slurry was prepared with good stability. The femtosecond laser etching and contour measurement method was adopted to analyze the polishing material removal rate of the CVD single crystal diamond workpiece. The surface damage of the workpiece polished with SiC abrasive grains is minimal, while the workpiece with diamond abrasive grains has the largest material removal rate. With an increase in abrasive grain size, the polishing material removal rate increases, but new surface scratches and pits can be introduced if the grain size is too large. Therefore, a grain size of 2.5 μm was selected to improve the surface quality. The surface roughness first decreases and then increases with the increase in polishing rotation speed. At a speed of 4000 rpm, the surface roughness reached its minimum with a relatively high material removal rate simultaneously. A series of CVD single crystal diamond scratching experiments were conducted with different scratching speeds, which proved that graphene oxide can help facilitate material surface micro-protrusion removal.

## 1. Introduction

The need for high-performance workpiece surfaces has been highly emphasized due to the increasingly strenuous working conditions in modern ultra-high-precision processes [1,2,3,4]. With the development of artificial diamond technology and the application of diamond in various fields, the surface quality of diamond has put forward increasingly higher requirements [5,6,7,8]. In the chemical mechanical polishing process of single crystal diamond, the oxidizing agent in the polishing solution reacts chemically with the carbon atoms on the surface of single crystal diamond to generate a relatively soft reaction layer. The reaction layer is removed under the mechanical action of the abrasive grains in the polishing solution. Due to the strong covalent bonds of diamond, the activation energy required to achieve oxidation is very high. The mechanical scrubbing of the abrasive grains breaks the C-C bonds of the diamond and forms a disordered layer. The deformed diamond obtains energy from the mechanical action and reduces the activation energy required for the chemical reaction to occur. In chemical mechanical polishing, mechanical action and chemical reaction cooperate to achieve the removal of diamond material. This method does not require complex experimental equipment, and the polished surface is quite smooth, which can realize a high material removal rate, and is a very promising polishing method for diamond polishing [9,10,11,12]. The high chemical inertness of diamond makes it less susceptible to chemical reactions with other substances.

The study of diamond polishing began at the beginning of the last century, and researchers have made numerous attempts to achieve high polishing efficiency and little damage [13,14,15]. Kubota et al. [16] applied polishing discs with highly uniformly set micron-sized diamond grits to polish the crystalline surface of single crystal diamond (100). The average surface roughness of 0.1–0.3 nm was achieved with appropriate processing parameters. Doronin et al. [17] applied electroplated diamond cast iron discs for staged mechanical polishing of single crystal diamond in different crystal orientations. The crystal orientation (100), which was easier to process, was first polished for material removal. The crystal orientation (110), which was more challenging to process, was then polished to improve surface roughness. The results show that the surface roughness of single crystal diamond is less than 0.5 nm.

Kühnle et al. [18] chose molten NaNO_3_ and KNO_3_ as oxidizing agents in their experimental study of the chemical mechanical polishing of diamond. In polishing the (100) crystalline surface, the roughness was reduced from 1.7 nm to about 0.2 nm after 0.5 h of polishing at a rate of 0.5 μm/h. The surface roughness was reduced from 4.1 nm to about 0.19 nm after 0.5 h of polishing the (111) crystalline surface. Wang et al. [19] reduced the melting point using a composite oxidant (LiNO_3_ + KNO_3_) to improve the material removal rate and surface roughness. The surface roughness of the diamond film was reduced from Ra 8–17 μm to 0.4 μm at 623 K, and the material removal rate could reach 1.7–2.3 mg/cm^2^/h. The use of molten salts as the oxidizing agent requires high temperature, which is generally higher than the melting point of the molten salts, and the melting points of NaNO_3_, KNO_3_, and KOH are 308 °C, 324 °C, and 360 °C, respectively. Therefore, the high-temperature environment during the polishing process will cause the polishing slurry to evaporate, which will cause environmental pollution as well as damage to the health of individuals. At the same time, the high temperature will cause thermal deformation of the polishing disk, which will affect the surface quality of the diamond after polishing.

To minimize the effect of temperature, researchers have started to search for low-temperature oxidants and replace the molten salts with strong oxidizing agents. Yuan et al. [20,21] used B_4_C powder with an average size of 2 μm as abrasive at 50 °C. Ten different polishing solutions were prepared using (NH_3_)_2_S_2_O_2_, H_2_O_2_, K_2_Cr_2_O_7_, KIO_4_, KMnO_4_, K_2_FeO_4_, CrO_3_, and Na_2_MoO_4_ as oxidizing agents to investigate the properties of chemical mechanical polishing solutions for the chemical vapor deposition of diamond films. It is confirmed that the oxidizing agent has a great influence on the polishing rate and surface quality in the CMP process, and the K_2_FeO_4_ shows great advantages in the CMP process. The highest material removal rate of 0.055 mg/h was obtained, with the best surface finish (~0.5 nm) and surface quality (no surface scratches or pits). Chemical mechanical polishing with a strong oxidizing agent polishing solution does not require particularly high temperatures, and the surface quality of the processed diamond is higher, but the effects of the evaporation of the polishing solution and the thermal deformation of the polishing disc could not be eliminated fundamentally.

Therefore, it is necessary to explore a new type of polishing solution for the chemical mechanical polishing of diamond. Graphene oxide (GO), as a derivative of graphene, containing hydroxyl, carboxyl, and other oxygen-containing groups, has better dispersibility and processability in solution and can be used as a substitute for graphene materials. There are a lot of oxygen-containing functional groups on the surface of graphene oxide. Under mechanical action, the graphene oxide polishing solution has high chemical activity, which can generate free radicals to promote the oxidation reaction on the processed surface. An oxide layer can be generated to promote material removal and improve surface roughness. In addition, graphene oxide has superior lubricity in high-speed polishing, which can improve the friction performance of the abrasive particles in polishing and the surface quality after polishing. Meanwhile, graphene oxide can also act as a coolant to improve the thermal impact of abrasive scratches during the polishing process and reduce the impact of thermal deformation of the polishing disk. Furthermore, the addition of graphene oxide as a polishing solution can also eliminate the effect of evaporation of the polishing solution, which is a green and environment-friendly polishing solution.

Polar groups such as hydroxyl, carboxyl, and epoxyl groups on the surface of graphene oxide can form a lubricating film on the friction surface with friction-reducing and anti-wear effects. Wu et al. [22] investigated the tribological properties of graphene oxide by dispersing graphene oxide in a base emulsion and conducting friction comparison experiments with the base emulsion. The coefficient of friction and abrasion depth of graphene oxide emulsion were decreased compared to base emulsion lubrication. It is mainly because the graphene oxide layer enters the area between the friction pairs to form a lubricating protective film, which can effectively reduce the wear of the two friction surfaces. Guo et al. [23] investigated the friction mechanism of graphene oxide. The results indicated that graphene oxide and SiO_2_ nanoparticles enhanced the lubricity and anti-wear properties of the friction film. It was concluded that the graphitization of graphene oxide nanosheets during the friction process facilitated the easy shear properties of the friction film, making the graphite oxide play a friction-reducing role. Meanwhile, compared with graphene, the polar functional groups on the surface of graphene oxide can be chemically modified according to different requirements, allowing graphene oxide to be suitable for more applications.

Chen et al. [24] created new composite abrasives for polishing silicon wafers. In order to increase the Ce ^3+^ content and the number of chemical reaction sites available during the polishing process, the proposed composite abrasive made use of graphene oxide’s unique structure. This accelerates the generation rate of the oxide layer on the silicon wafer surface, which increases the material removal rate and improves the surface quality. Huang et al. [25] developed a novel polishing process for single crystal SiC by using a graphene oxide/diamond hybrid nanosuspension. Both the polishing surface quality and polishing efficiency were improved, which is due to the synergistic effect of the oxidation and lubrication generated by graphene oxide. Liu et al. [26] conducted single crystal aluminum oxide sapphire wafers lapping experiments using graphene compound slurry, including graphene oxide, reduced graphene oxide, ML-RGO (laminate), and MF-RGO (few-layer). A low surface roughness and high material removal rate can be achieved with the graphene oxide compound slurry, when the synergistic equilibrium of mechanical removal and chemical reaction forms. Liu et al. [27] also developed a chemically grafted polyurethane/graphene ternary slurry for SiC wafer polishing. The material removal rate, surface roughness, and surface roughness improvement rate were studied, and a better performance of the ternary slurry was verified.

In previous studies, the polishing process of single crystal diamond with GO-enhanced hybrid slurry focused on the surface quality, while the removal rate of single crystal diamond material was less studied. Therefore, in this paper, the polishing material removal rate of CVD single crystal diamond workpieces with GO-enhanced hybrid slurry was investigated.

## 2. The Experimental Materials and Setup

### 2.1. The Experimental Materials

The CVD single crystal diamond (100) workpieces with the size of 3 mm × 3 mm × 1 mm are provided by Ningbo Crysdiam Technology Co., Ltd. (Ningbo, China), which has the hardness of 8300–12,000 HV, the density of 3.52 g/cm^3^, the Young’s modulus of 1000 Gpa, the bending strength of 2.0 Gpa, the thermal conductivity of 1100 W/m K [19]. The monolayer GO used in this study is provided by Jiangsu Xianfeng nanomaterials Technology Co., Ltd. (Nanjing, China), which has a thickness of 0.8~1.2 nm, a sheet diameter of 0.5~5 μm, a purity of 99%, a monolayer rate of 99%.

### 2.2. Polishing Experimental Setup

**Figure 1:**
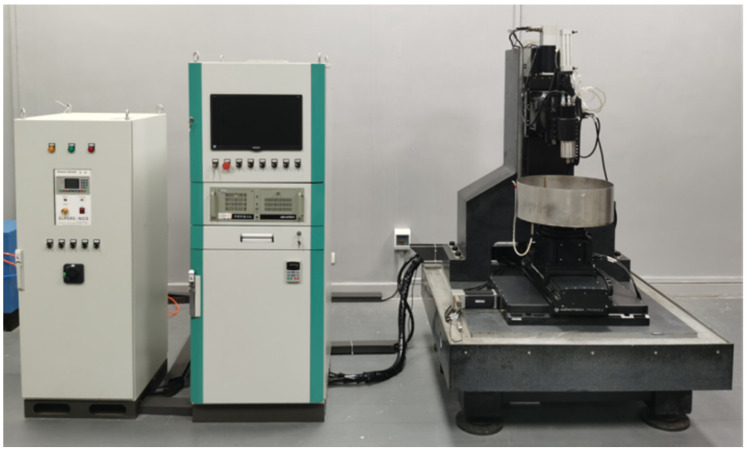
The five-axis CNC polishing machine.

The single crystal diamond polishing experiments with GO hybrid slurry were carried out on a five-axis CNC polishing machine, as shown in Figure 1. The diamond workpieces were fixed on a polishing head with epoxy resin. The glass polishing discs were fixed on the fixture.

### 2.3. The Preparation of GO-Enhanced Diamond Hybrid Slurry

The analytical balance with a measuring range of 100 g and an accuracy of 0.1 mg was used to weigh graphene oxide, deionized water, and diamond grain slurry. A constant temperature magnetic stirrer was used to stir the GO nanosheets aqueous solution and GO-enhanced diamond hybrid slurry. The ultrasonic cleaner was used to evenly disperse graphene oxide nanosheets in deionized water.

**Figure 2:**
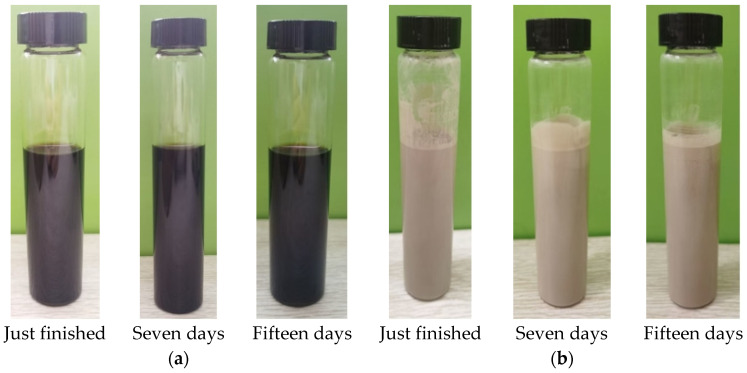
The stability of (**a**) the GO nanosheets aqueous solution and (**b**) the GO-enhanced diamond hybrid slurry.

An aqueous solution with a mass percentage of 0.49 wt% was prepared by weighing 0.2 g of GO powder and 40 g of deionized water using an analytical balance. The GO nanosheets and deionized water were mixed with a magnetic stirrer for 30 min. Then, the magnetically stirred aqueous solution was placed in an ultrasonic cleaner for ultrasonic dispersion (the water in the water tank should be guaranteed to be greater than one-half of the depth of the water tank). The time of each ultrasonication was 30 min. Because GO will be reduced above 38 °C, the temperature during the preparation process is controlled at 30 °C. Stirring and ultrasonic dispersion were repeated several times until the GO nanosheets were uniformly dispersed in deionized water. Finally, the stability of the aqueous solution of GO nanosheets was observed after settling. Figure 2a shows the aqueous solution of GO nanosheets after just finished, seven days, and fifteen days. The agglomeration, settling delamination, or other phenomena did not occur in the prepared GO aqueous solution.

The GO-enhanced diamond hybrid slurry with a mass percentage of 0.1 wt% was dispensed by 20 g of GO nanosheets aqueous solution and 80 g of diamond slurry with a particle size of 2.5 μm. The weighed GO nanosheets aqueous solution and diamond slurry were stirred in a magnetic stirrer at 500 rpm for 30 min. Then, the hybrid slurry was performed with ultrasonic dispersion for 30 min. The stirring and ultrasonic dispersion were repeated several times alternately until the GO nanosheets were homogeneously dispersed in the hybrid slurry. Finally, the stability of GO green-enhanced diamond hybrid slurry was observed after static settling. Figure 2b shows the GO-enhanced diamond hybrid slurry after just finished, seven days and fifteen days. The aggregation, sedimentation, delamination, or other phenomena did not occur as well in the prepared GO-enhanced diamond hybrid slurry, so it was stable and reasonable.

### 2.4. Characterization Setup and Methods

The high hardness and chemical inertness of diamond makes it difficult to be machined. It is difficult to characterize the material removal rate of the polishing process because the material removal rate is very low. The conventional weighing method weighs the workpieces before and after polishing separately to calculate the material removal rate. However, impurities are inevitably introduced to the surface of the diamond workpiece during the polishing and weighing process. Furthermore, the precision of the analyzing balance and the operation process are very demanding. Therefore, the traditional weighing method is not suitable for the characterization of diamond material removal rate.

The contour measurement method was adopted to calculate the material removal rate (MRR) of diamond. Grooves with a certain depth were machined by a femtosecond laser in the middle area of the workpiece surface. The depth of the grooves before and after polishing was measured using a 3D measuring laser microscope (OLYMPUS LEXT OLS4100, Olympus Corporation, Tokyo, Japan). Before surface morphology measuring, the ultrasonic cleaner was used to clean the workpiece surface with alcohol and deionized water to remove the impurities caused by laser process and polishing. The difference in depth of the grooves was used to represent the thickness of the material removed. Equation (1) was used to calculate the MRR.

MRR = δ/t,(1)

where δ is the difference in depth before and after polishing and t is the polishing time.

**Figure 3:**
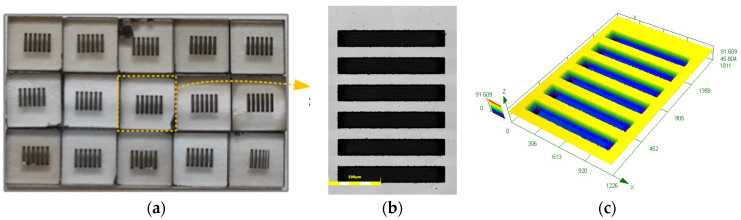
The surface of single crystal diamond after femtosecond laser processing: (**a**) The single crystal diamond workpiece; (**b**) 2D morphology of grooves; (**c**) 3D morphology of grooves.

The surface of single crystal diamond after femtosecond laser processing can be seen in Figure 3. Each diamond surface was machined with 6 grooves with a depth of 40 μm, a width of 140 μm, and a length of 1 mm. The average value of the height difference of the 6 grooves was taken to calculate the MRR.

## 3. The Polishing Characteristics of GO-Enhanced Hybrid Slurry

### 3.1. The Polishing Characteristics with Different Abrasive Grain Type

The abrasive grain is one of the main components of the polishing slurry and determines the mechanical action and the material removal rate after polishing. Among them, abrasive grain type and its hardness are the main influencing factors for polishing efficiency and polishing surface quality. The polishing process parameters used are a polishing depth of 0.1 mm, a polishing rotation speed of 4000 rpm, and a polishing duration of 70 min.

Four types of abrasive grains with different hardness were selected for the experimental comparison. The microhardness and grain size parameters of the abrasive grains are shown in Table 1. Diamond, silicon carbide, alumina, and cerium dioxide slurry with GO mass percentage of 0.1 wt% were configured, respectively.

#### 3.1.1. Polishing Surface Quality

**Figure 4:**
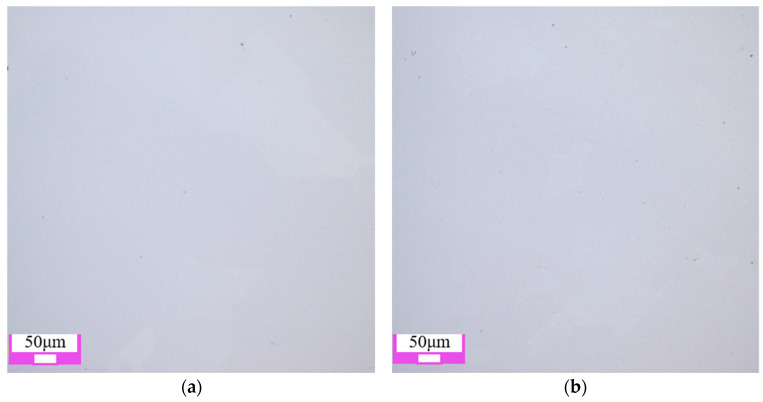

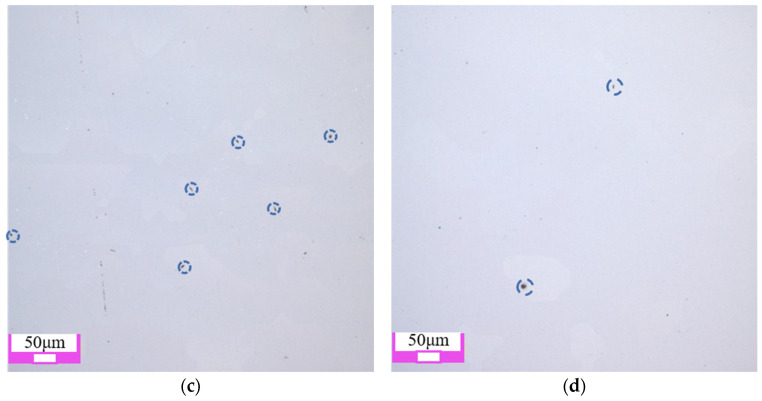
Polishing surface morphology of single crystal diamond with different abrasive grain types: (**a**) diamond abrasive grain; (**b**) SiC abrasive grain; (**c**) Al_2_O_3_ abrasive grain; (**d**) CeO_2_ abrasive grain.

The two-dimensional surface morphology of single crystal diamond is shown in Figure 4. Among the four types of abrasive grains, the surface damage of the workpiece after polishing with SiC abrasive grains is minimal, and the original defects such as pits and scratches on the surface of the workpiece are basically removed. The surface quality after polishing with diamond grains is slightly lower than that after polishing with SiC grains, as shown in Figure 4a,b. When Al_2_O_3_ and CeO_2_ abrasive grains are used, there are still some pits on the polished single crystal diamond surface, as shown in Figure 4c,d. The main reason is that single crystal diamond has high hardness and chemical inertness, and the hardness of Al_2_O_3_ and CeO_2_ abrasive grains is low, which cannot completely remove the original defects on the surface of single crystal diamond.

**Figure 5:**
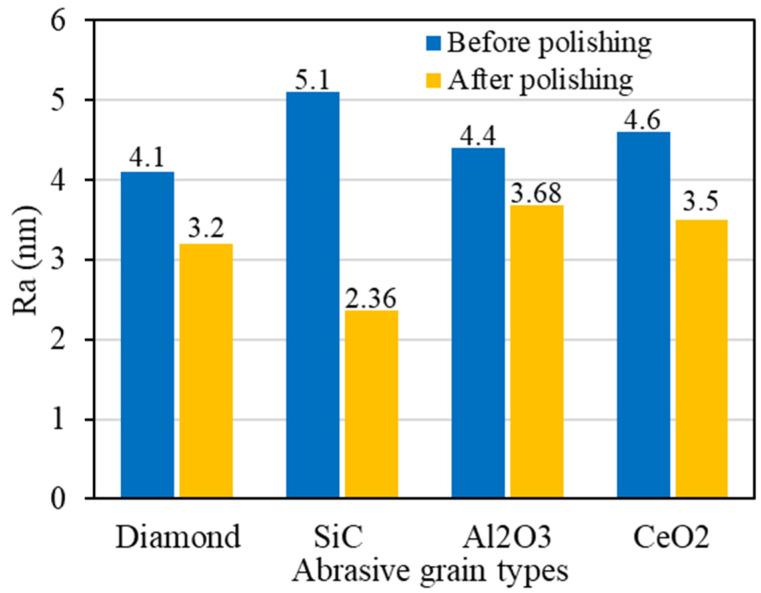
The surface roughness of single crystal diamond after polishing with different abrasive grain types.

The surface roughness of the polished single crystal diamond workpiece is shown in Figure 5. Under the mechanical action of abrasive grains and the chemical action of graphene oxide, the micro-peaks on single crystal diamond surface can be effectively improved. The surface roughness of the workpiece can be improved to a certain extent. When SiC abrasive grains are used, the surface roughness of the workpiece decreases from Ra 5.1 nm to Ra 2.36 nm, which is the most significant reduction. In contrast, the surface roughness decreases less when using diamond, Al_2_O_3_, and CeO_2_ abrasive grains.

#### 3.1.2. Polishing Material Removal Rate

**Figure 6:**
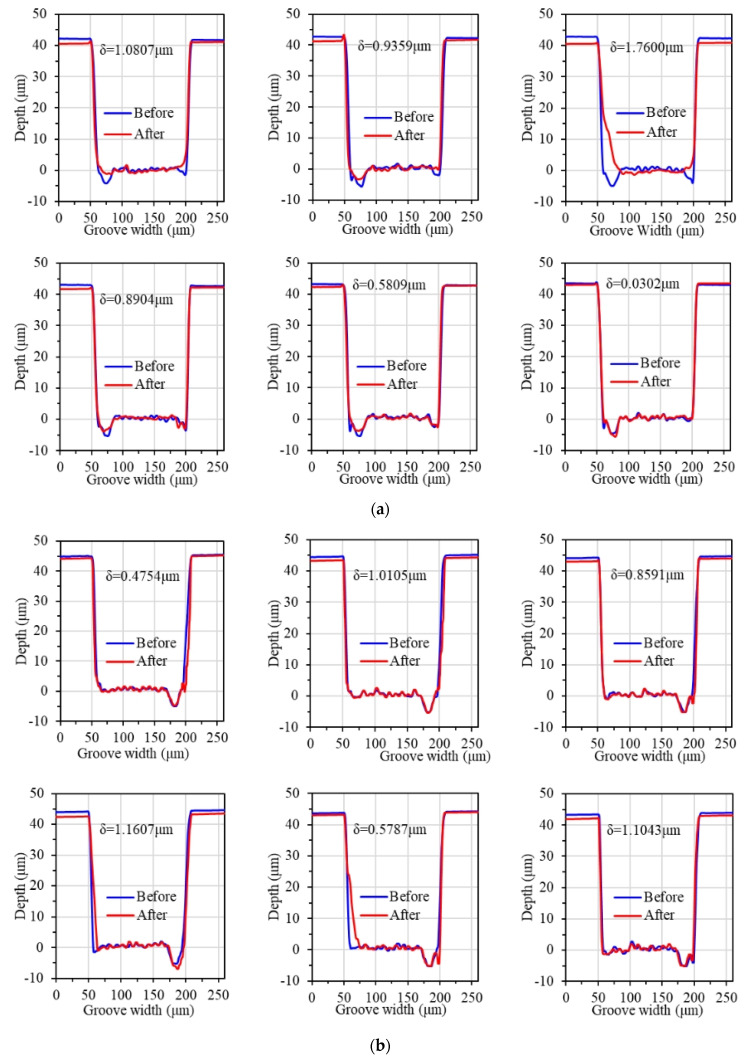

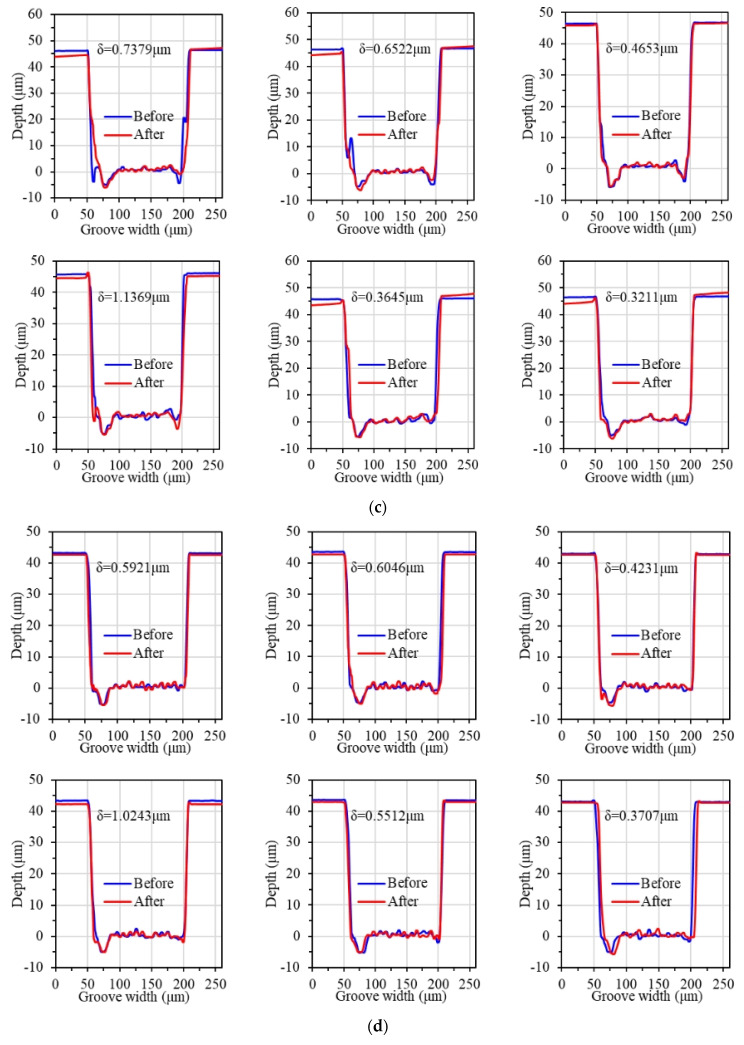
Material removal depth with different abrasive grain types: (**a**) diamond abrasive grain; (**b**) SiC abrasive grain; (**c**) Al_2_O_3_ abrasive grain; (**d**) CeO_2_ abrasive.

**Figure 7:**
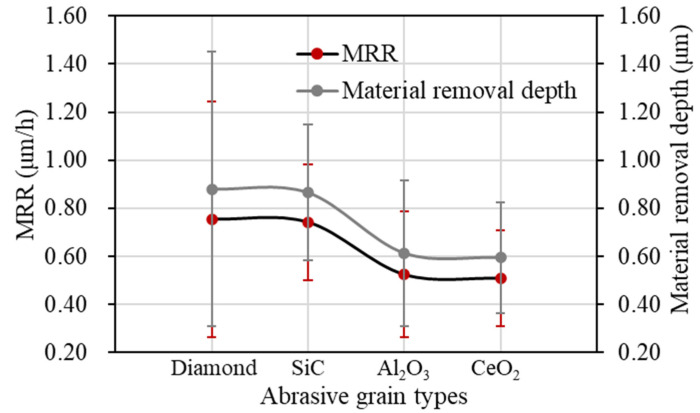
Material removal rate with different abrasive grain types.

The depths of material removal after polishing for different abrasive grain types are shown in Figure 6 and Figure 7, respectively. It can be seen that as the hardness of the abrasive grains decreases, the material removal rate also decreases. Diamond abrasive grains remove more material per unit time and have the largest material removal rate of 0.754 μm/h. Cerium oxide abrasive grains have the lowest hardness among the four types, and, therefore, have the lowest material removal rate of 0.509 μm/h.

### 3.2. The Polishing Characteristics with Different Abrasive Grain Sizes

The abrasive grain size has a great influence on the polished surface quality and material removal rate of single crystal diamond. When the grain size is larger, the material removal rate is higher. However, larger abrasive grains will introduce new scratches on the polished surface, then the surface quality after polishing will be poor. When the grain size is small, the surface quality can be improved. However, the contact area between the grain and the surface is small, which is not conducive to improving the polishing efficiency. Therefore, it is necessary to select the appropriate abrasive grain size to achieve good surface quality and material removal rate. In order to investigate the effect of different grain sizes on polishing effect, four kinds of diamond grains with different grain sizes were selected to carry out chemical mechanical polishing comparison experiments with a polishing depth of 0.1 mm, a polishing rotation speed of 4000 rpm, and a polishing duration of 70 min, as shown in Table 2.

#### 3.2.1. Polishing Surface Quality

**Figure 8:**
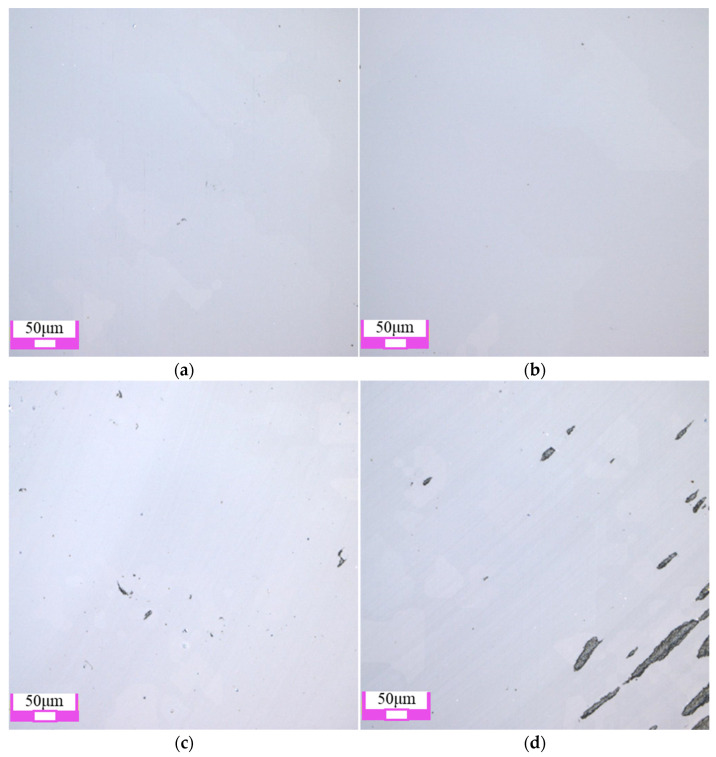
Polishing surface morphology of single crystal diamond with different abrasive grain sizes: (**a**) 0.5 μm; (**b**) 2.5 μm; (**c**) 5 μm; (**d**) 9 μm.

As shown in Figure 8, when the grain size is small (0.5 μm and 2.5 μm), the generated softer reaction layer is removed under the friction and impact of the abrasive grains. A smooth surface is obtained, and the surface quality is significantly improved. When diamond grains with the size of 5 μm are used, new pits are formed on the single crystal diamond surface. When the abrasive grain size reached 9 μm, the polished surface not only appeared pits, but also appeared wider scratches. Its surface quality has deteriorated significantly. This is mainly because, in the process of chemical mechanical polishing, the increase in grain size enhances its mechanical effect. The abrasive is directly applied on the diamond workpiece surface after the chemical reaction layer is removed, and larger grains can lead to more severe subsurface damage.

**Figure 9:**
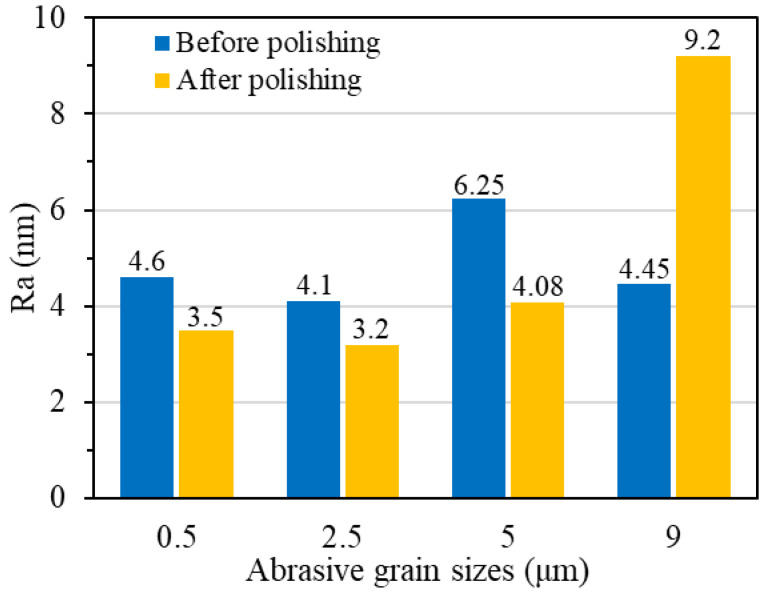
Polishing surface roughness of single crystal diamond with different abrasive grain sizes.

As shown in Figure 9, with the increase in abrasive grain size, the surface roughness of polished single crystal diamond surface also increases. When the abrasive grain size reaches 9 μm, the surface roughness increases from 4.45 nm to 9.2 nm. This corresponds to the surface morphology of the polished single crystal diamond surface in Figure 8d.

#### 3.2.2. Polishing Material Removal Rate

**Figure 10:**
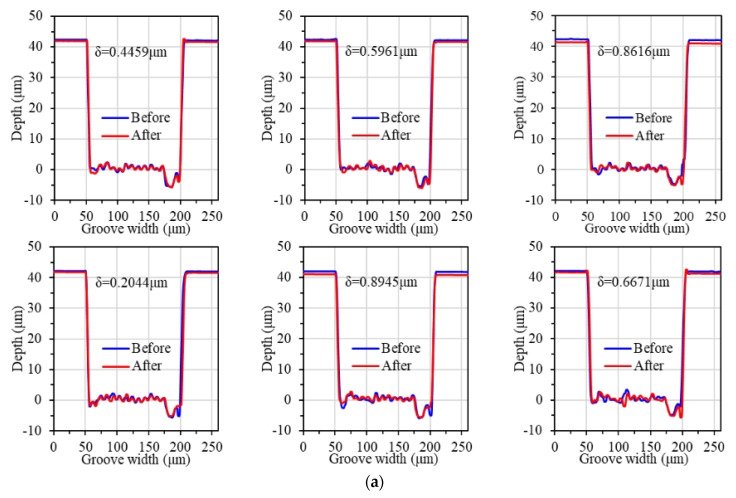

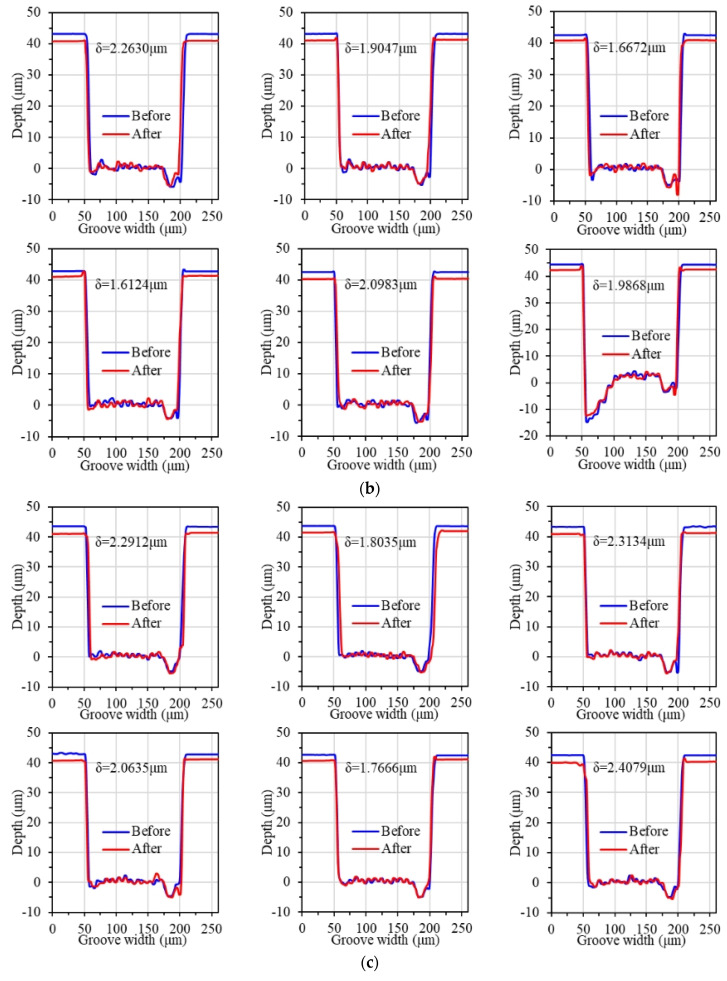
Material removal depth with different abrasive grain sizes: (**a**) 0.5 μm; (**b**) 5 μm; (**c**) 9 μm.

**Figure 11:**
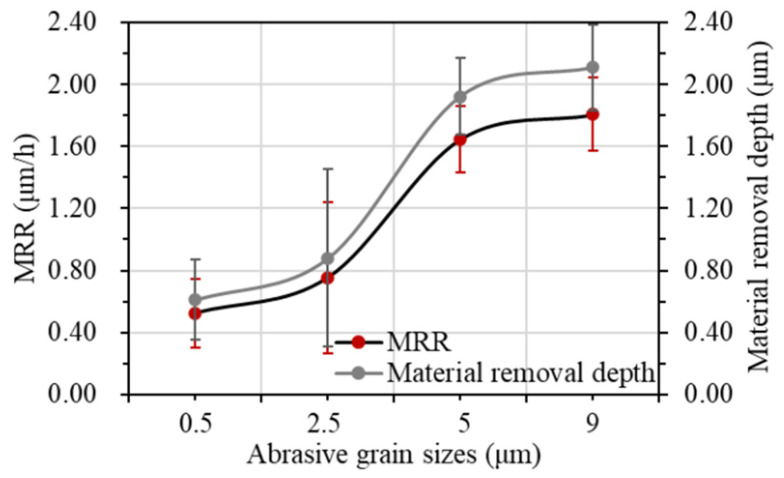
Material removal rate with different abrasive grain sizes.

As shown in Figure 6a, Figure 10 and Figure 11, the material removal rate increases gradually with the increase in grain size. When the grain size is 9 μm, the maximum material removal rate is obtained as 1.807 μm/h. When the grain size is 0.5 μm, the material removal rate reaches the lowest at 0.524 μm/h. When the grain size exceeds 5 μm, the material removal rate increases slowly. The material removal rate with the grain size of 9 μm is only 9.71% higher than that of 5 μm.

Based on the above analysis, when using 0.5 μm and 2.5 μm diamond abrasive grains for polishing, most of the original surface defects can be removed within 70 min. The surface quality can be obviously improved. However, the polishing material removal rate with 2.5 μm abrasive grains is 1.4 times than that with 0.5 μm abrasive grains. When the abrasive grain size reaches more than 5 μm, although the material removal rate is high, the surface quality after polishing is poor. Considering the surface quality and material removal rate, the diamond abrasive grains with the size of 2.5 μm were used for the single crystal diamond chemical mechanical polishing.

### 3.3. The Polishing Characteristics with Different Rotation Speeds

In the polishing process of single crystal diamond, the polishing rotation speed is an important influencing factor for the polishing efficiency. The polishing rotation speed can change the kinetic energy of the abrasive grains, making the abrasive grains produce effective shear force, which helps to improve the mechanical action in the polishing process. At the same time, the change in polishing rotation speed affects the temperature of the polishing area. The higher the polishing rotation speed, the greater the fluid dynamic pressure between the single crystal diamond workpiece and the polishing disk, which generates a higher temperature. It can promote the oxidation reaction of the oxidizer and produce more activated carbon atoms, thus further increasing the material removal rate.

It is necessary to study the influence law of polishing rotation speed on the chemical mechanical polishing of single crystal diamond. Four different polishing rotation speeds were set. The polishing duration was 70 min. The polishing slurry with graphene oxide of 0.1 wt% and diamond abrasive grain size of 2.5 μm was configured, and the specific parameters are shown in Table 3.

#### 3.3.1. Polishing Surface Quality

**Figure 12:**
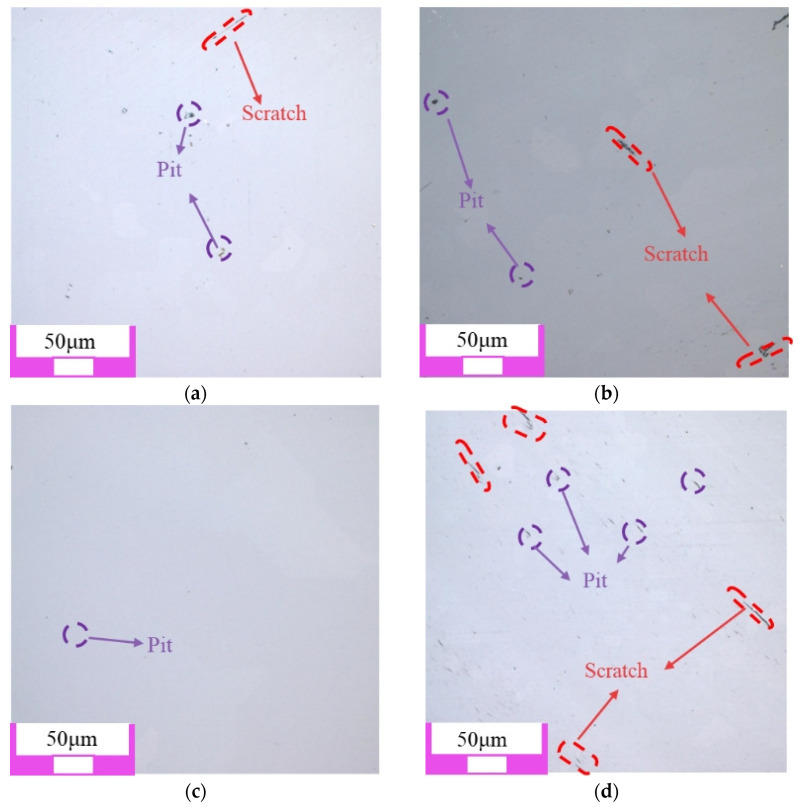
Polishing surface morphology of single crystal diamond with different polishing rotation speeds: (**a**) 2000 rpm; (**b**) 3000 rpm; (**c**) 4000 rpm; (**d**) 5000 rpm.

**Figure 13:**
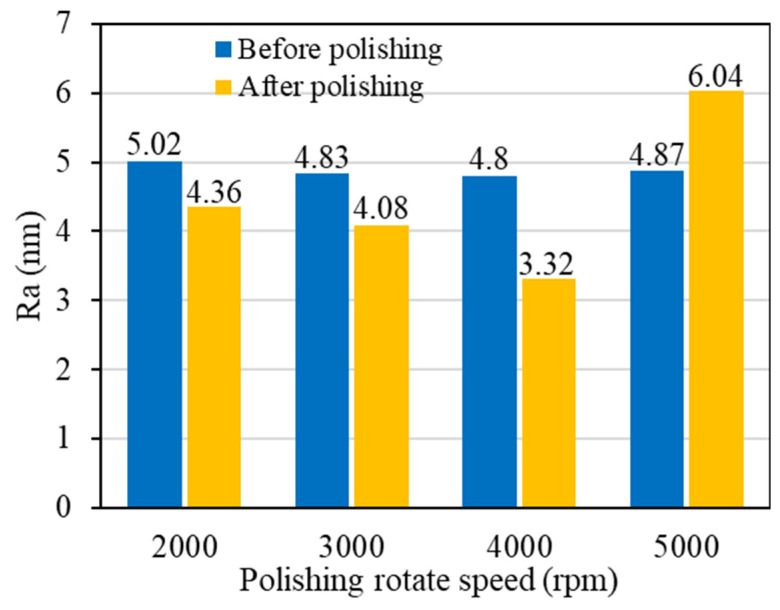
Polishing surface roughness of single crystal diamond with different polishing rotation speeds.

The polishing pressure and the polishing duration were set to 30 N and 70 min, respectively. As shown in Figure 12 and Figure 13, when the polishing rotation speed increased from 2000 rpm to 4000 rpm, the surface roughness of the single crystal diamond gradually decreased, and the surface quality of the workpiece improved. At a speed of 4000 rpm, the surface roughness reached its minimum, Ra 3.32 nm. This is because the increased polishing rotation speed led to more frequent interactions between the abrasive grains and the workpiece surface per unit time, allowing the reaction layer on the workpiece surface to be removed promptly. However, when the polishing rotation speed was increased to 5000 rpm, the surface roughness increased from Ra 4.87 nm to Ra 6.04 nm. The abrasive grains scratched directly on the workpiece surface before the GO-induced degraded layer formed.

#### 3.3.2. Polishing Material Removal Rate

**Figure 14:**
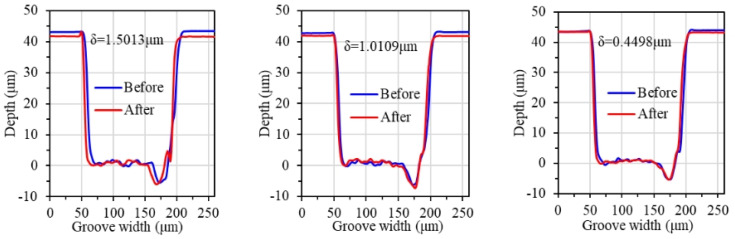

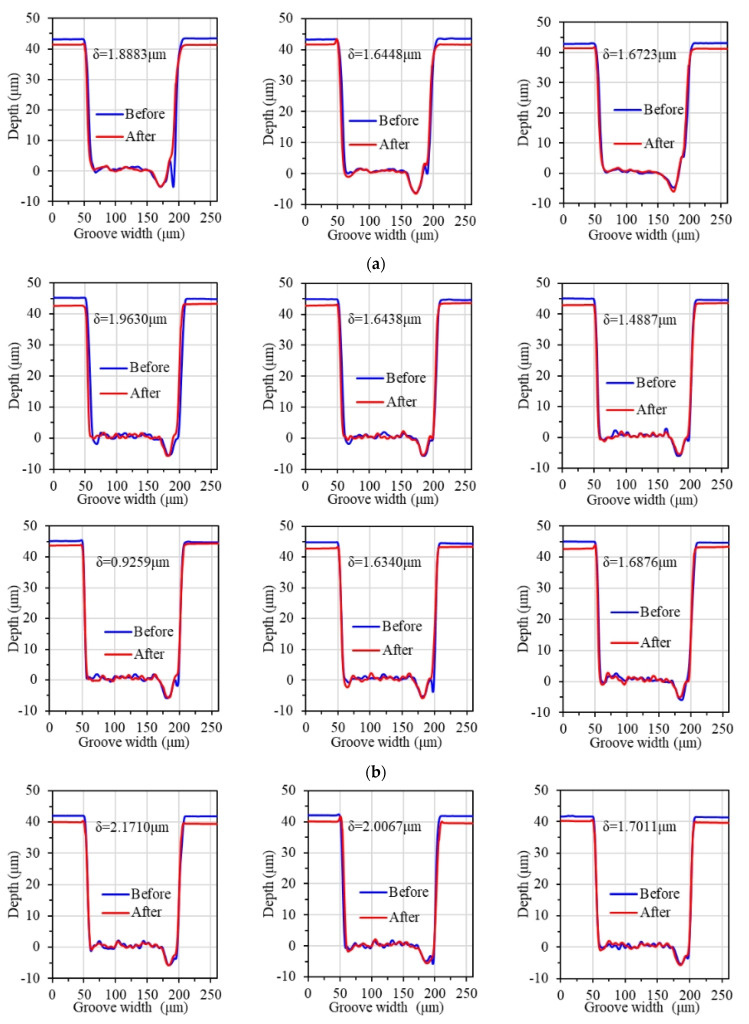

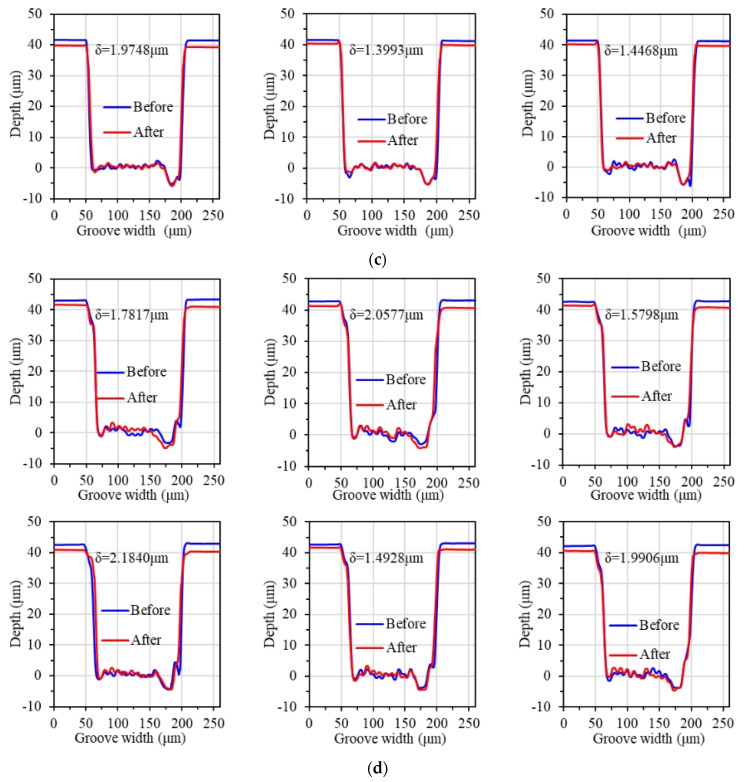
Material removal depth at different polishing rotation speeds: (**a**) polishing rotation speed of 2000 rpm; (**b**) polishing rotation speed of 3000 rpm; (**c**) polishing rotation speed of 4000 rpm; (**d**) polishing rotation speed of 5000 rpm.

**Figure 15:**
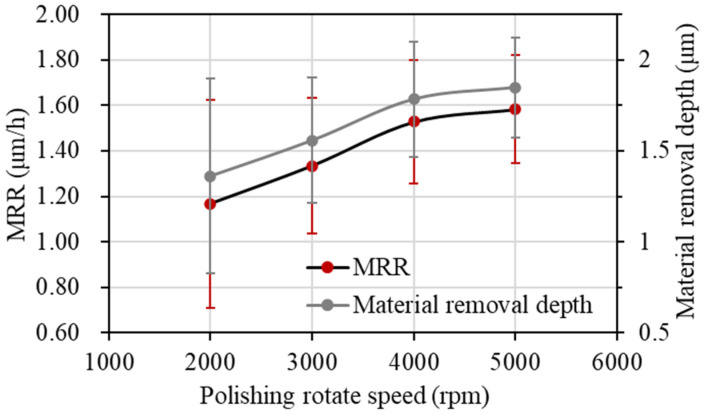
Material removal rate at different polishing rotation speeds.

As shown in Figure 14 and Figure 15, the material removal rate increases gradually with the increase in polishing rotation speed. When the polishing rotation speed is increased from 2000 rpm to 4000 rpm, the material removal rate increases faster, and reaches 1.529 μm/h at 4000 rpm. On one hand, with the increase in the polishing rotation speed, the mechanical action of the abrasive grain is enhanced, and more surface micro bumps are removed, so the material removal rate increases with the increase in polishing speed. On the other hand, the increase in polishing speed makes the temperature of the contact area increase, which can promote the chemical reaction of the oxidizer in the polishing slurry to produce more activated atoms, which is conducive to further improving the material removal rate. When the polishing rotation speed continues to increase to 5000 rpm, the material removal rate is not significantly improved, and reaches 1.584 μm/h. This is mainly because the high polishing rotation speed increases the centrifugal effect of the polishing slurry, so that a large amount of polishing slurry is thrown out of the contact area. The number of abrasive grains in the contact area is reduced.

## 4. The Single Crystal Diamond Scratching Experiments with GO Slurry

### 4.1. Experimental Setup

To further investigate the performance of GO in the surface material removal of single crystal diamond, mechanical scratching experiments of single crystal diamond were conducted with 0.1 wt% GO slurry. The experiments were performed on the Multi-functional Material Surface Property Tester (MFT-4000, Lanzhou Huahui Instrument Technology Co., Ltd. (Lanzhou, China)), as shown in Table 4. The contact mode of the friction pair in the experiment was in the form of “point-face contact”. The single crystal diamond workpiece was held on the working table to conduct reciprocating linear motion. The friction ball was embedded in a pillar. The friction force and coefficient could be measured directly by the tester.

To better analyze the performance of GO during the polishing process, six conditions with different velocities were set in the scratching experiments, with a friction length of 5 mm and a friction time of 20 min. The experimental parameters are shown in Table 5. To ensure the reliability of the experimental results, all workpieces and friction balls were ultrasonically cleaned with alcohol for 10 min before the experiment.

### 4.2. Experimental Results and Analysis

**Figure 16:**
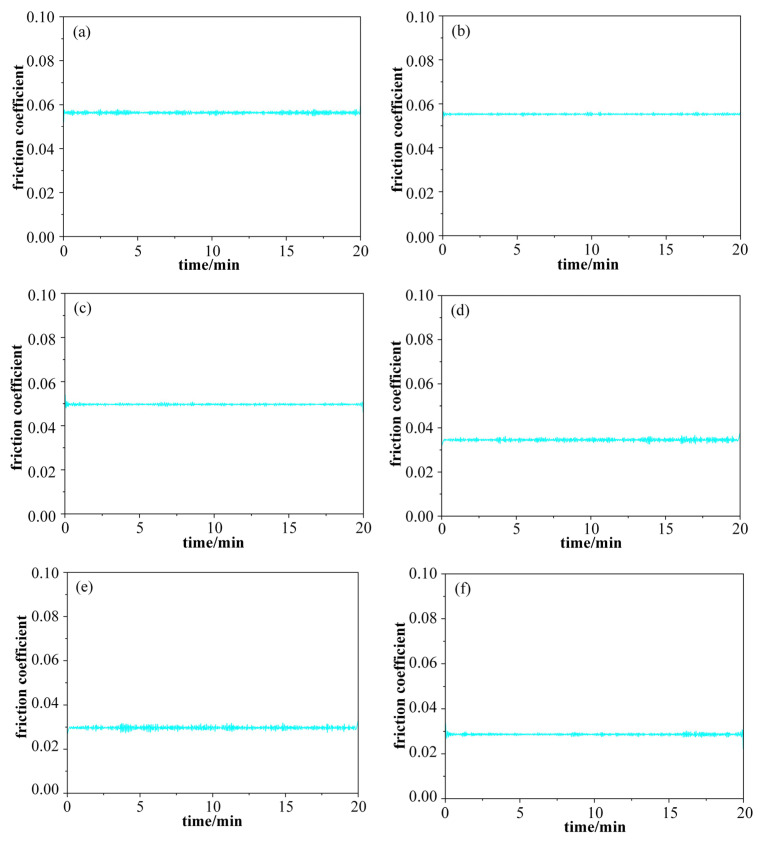
The friction coefficient curves under different working conditions: (**a**) 100 mm/min for dry friction; (**b**) 150 mm/min for dry friction; (**c**) 200 mm/min for dry friction; (**d**) 100 mm/min with GO; (**e**) 150 mm/min with GO; (**f**) 200 mm/min with GO.

**Figure 17:**
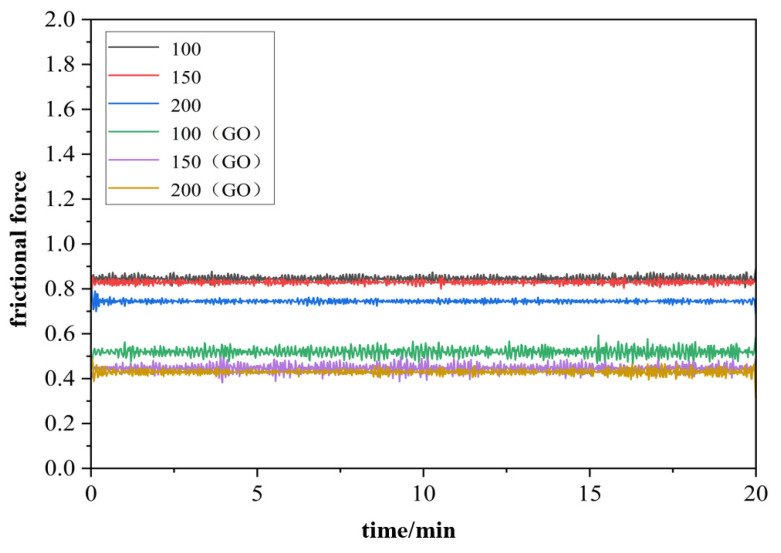
The friction force under different rotation speeds.

**Figure 18:**
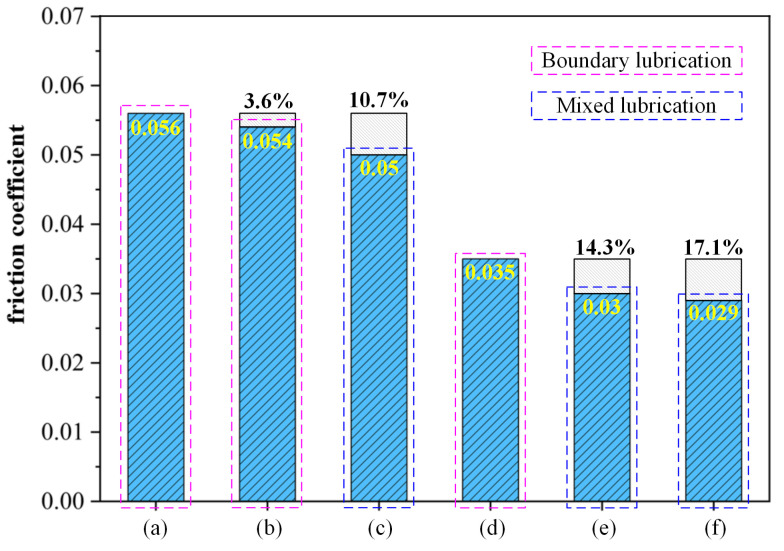
The friction coefficient under different speeds: (**a**) 100 mm/min for dry friction; (**b**) 150 mm/min for dry friction; (**c**) 200 mm/min for dry friction; (**d**) 100 mm/min with GO; (**e**) 150 mm/min with GO; (**f**) 200 mm/min with GO.

At a load of 15 N, six working conditions were set up with moving speeds of 100 mm/min (dry friction), 150 mm/min (dry friction), 200 mm/min (dry friction), 100 mm/min (0.1 wt%GO), 150 mm/min (0.1 wt%GO), and 200 mm/min (0.1 wt%GO). The curves of friction coefficient versus time and friction force versus time for each experimental condition were obtained as shown in Figure 16 and Figure 17, respectively. The average friction coefficients for each experimental condition were counted, as shown in Figure 18.

From the Stribeck curve, the friction coefficient of mixed lubrication is less than that of boundary lubrication [22]. In the initial stage of scratching, there are many small peaks and valleys in the contact area of the workpiece, so the friction coefficient is larger. It is considered a stage of boundary lubrication. As the scratching process progresses, the surface micro-peaks are gradually removed, and the contact area becomes smooth. The friction coefficient decreases. It is considered a stage of mixed lubrication. Therefore, the decrease in friction coefficient means the surface micro-protrusions are removed. In this study, it is assumed that the lubrication mechanism changes from boundary lubrication to mixed lubrication when the friction coefficient is reduced by more than 10%.

The friction coefficient and scratching force decrease as the scratching speed increases for a constant load, as shown in Figure 16 and Figure 18. The friction coefficient decreases even faster with GO slurry. As shown in Figure 18c, for dry conditions, the friction coefficient was reduced by more than 10%, when the moving speed reached 200 mm/min. However, with the addition of GO slurry, the friction coefficient decreased by more than 10% when the moving speed reached 150 mm/min. Therefore, the addition of GO is helpful in removing micro-protrusions on the surface of single crystal diamond. The possible reason is that there are a large number of unsaturated carbon atoms on the surface of single crystal diamond micro-protrusions, which are easier to oxidize with GO, thus promoting material removal.

The friction coefficient with GO was lower than that in dry friction conditions. Even in the mixed lubrication stage, the friction coefficient was 0.050 under 200 mm/min in the dry friction condition, while the friction coefficient was 0.029 after adding GO, which was 42% lower than that of the dry friction condition. The reason might be the good lubrication performance of GO, which improved the friction condition between the friction pair. The GO nanosheets can access the surface of the friction pair during the scratching process, forming a lubrication film on the surface between the friction ball and the workpiece.

## 5. Conclusions

The study investigates the preparation of graphene oxide-enhanced hybrid slurry and its polishing characteristic on CVD single crystal diamond workpieces. The research results and main conclusions are as follows:

(1) The GO slurry and GO-enhanced hybrid slurry with diamond, silicon carbide, alumina, and cerium dioxide abrasive grains were prepared with a magnetic stirring technique and ultrasonic dispersion technique. The stability of the GO-enhanced hybrid slurry was observed, and agglomeration, settling delamination, or other phenomena did not occur in the prepared hybrid slurry.
(2) Diamond, silicon carbide, alumina, and cerium dioxide abrasive grains with different hardness were selected for single crystal diamond polishing experiments with GO. The surface damage of the workpiece polished with SiC abrasive grains is minimal. Its surface roughness decreased to Ra 2.36 nm, which is the most significant. The surface quality polished with diamond grains is slightly lower. The workpiece with diamond abrasive grains has the largest material removal rate of 0.754 μm/h, while it has the lowest material removal rate of 0.509 μm/h with cerium oxide abrasive grains.
(3) The single crystal diamond polishing surface quality and material removal rate results were compared with different abrasive grain sizes. For small grain sizes of 0.5 μm and 2.5 μm, the surface quality can be obviously improved. However, the polishing material removal rate with 2.5 μm abrasive grains is 1.4 times higher than that with 0.5 μm abrasive grains. When the abrasive grain size reaches more than 5 μm, new pits and wider scratches are formed. Therefore, the diamond abrasive grains with the size of 2.5 μm were used for the single crystal diamond chemical mechanical polishing.
(4) The polishing surface roughness of the single crystal diamond first decreases and then increases with the increase in polishing rotation speed. At a speed of 4000 rpm, the surface roughness reached its minimum of Ra 3.32 nm. The material removal rate increases gradually with the increase in polishing rotation speed and reaches 1.529 μm/h at 4000 rpm. When the polishing rotation speed continues to increase to 5000 rpm, the material removal rate is not significantly improved.
(5) The single crystal diamond scratching experiments with GO slurry were conducted. As the scratching speed increases, the friction coefficient and scratching force decrease. However, the friction coefficient decreases faster with GO slurry, because the oxidation of GO is helpful to remove micro-protrusions on the surface of single crystal diamond.

## Figures and Tables

**Table 1:** The hardness and particle size of several types of abrasive grains.

Grain Types	Diamond	SiC	Al_2_O_3_	CeO_2_
Moh’s hardness	10	9.2~9.6	9	7
Grain size (μm)	2.5	3	3	3

**Table 2:** The diamond abrasive grain size parameters.

Parameters	Values			
Abrasive grain size (μm)	0.5	2.5	5	9
Abrasive grain type	Diamond	Diamond	Diamond	Diamond

**Table 3:** Different polishing rotation speed experimental parameters.

No.	a	b	c	d
GO content (wt%)	0.1	0.1	0.1	0.1
Abrasive grain size (μm)	2.5	2.5	2.5	2.5
Polishing pressure (N)	30	30	30	30
Polishing rotation speed (rpm)	2000	3000	4000	5000

**Table 4:** The parameters of Multi-functional Material Surface Property Tester.

Performances	Parameters
Type	MFT-4000
Manufacturers	Lanzhou Huahui Instrument Technology Co., Ltd.
Load range	0.5~300 N (Accuracy 0.25 N, automatic continuous loading)
Loading speed	1~100 N/min
Stroke	5~40 mm
Lifting height	20 mm
Reciprocation speed	1~240 mm/min (Accuracy 0.1 mm/min)
Contact type	Point-face
Friction fixture	Ball (Diameter 3 mm, 4 mm, 5 mm, 6 mm)

**Table 5:** The parameters of the friction and wear experiment.

Parameters	Value
Load (N)	15
Reciprocation speed (mm/min)	100, 150, 200
Distance (mm)	5
Time (min)	20

## Data Availability

The original contributions presented in this study are included in the article. Further inquiries can be directed to the corresponding authors.
